# The Effect of Diabetes Mellitus on Cardiac Remodeling and Overall Clinical Outcomes in Patients With Acute Myocardial Infarction: A Single-Center Retrospective Study in Saudi Arabia

**DOI:** 10.7759/cureus.49281

**Published:** 2023-11-23

**Authors:** Ammar A Albakistani, Ahmed A Alqerafi, Yasir O Marghalani, Rami S Alasmari, Anas M Alswat, Sereen A Banjar, Reem F Allam, Mohamed E Ahmed, Atif Alzahrani

**Affiliations:** 1 College of Medicine, King Abdullah International Medical Research Center, Jeddah, SAU; 2 College of Medicine, King Saud Bin Abdulaziz University for Health Sciences, Jeddah, SAU; 3 College of Sciences and Health Professions, King Saud Bin Abdulaziz University for Health Sciences, Jeddah, SAU; 4 Department of Cardiac Sciences, King Faisal Cardiac Center, Jeddah, SAU

**Keywords:** myocardial infarction, heart failure, left ventricular remodeling, diabetes mellitus, st-elevation myocardial infarction

## Abstract

Background: Diabetes mellitus (DM) is a major chronic condition that is considered a strong indicator of poor cardiovascular outcomes, such as recurrent infarction and heart failure (HF), in individuals with acute myocardial infarction (AMI). However, the concept of left ventricular remodeling (LVR) following AMI in DM patients is not well understood and studied in Saudi Arabia. Thus, the aim of this study is to assess the association between LVR and DM in patients presenting with ST-elevation myocardial infarction (STEMI) who had reperfusion therapy with optimal medical therapy after percutaneous coronary intervention (PCI).

Methods: In this retrospective cohort study, 171 patients diagnosed with AMI who visited King Faisal Cardiac Center in King Abdulaziz Medical City, National Guard Hospital, Jeddah, Saudi Arabia, were chosen via the convenience sampling method. The study included patients with AMI who received echocardiograms upon admission and during a follow-up period of six to 12 months. The patients were divided into two groups based on their diabetic status: diabetic (DM) and non-diabetic (non-DM). To collect the data, trained medical students supervised by the principal investigator used the patients’ medical records.

Results: The study showed that DM patients were more likely to have a history of hypertension, dyslipidemia, smoking, and stress hyperglycemia and had a higher hospitalization rate compared to the non-DM group. Although there was no statistically significant difference (p=0.253), both groups had a higher incidence of the left main trunk and/or left anterior descending artery affected. Regarding the echocardiographic finding, there were no significant differences between the two groups in terms of left ventricular ejection fraction, left ventricular internal diameter at end-diastole, left ventricular internal diameter at end-systole, and interventricular septum thickness.

Conclusion: This paper suggests that there is no significant correlation between DM and non-DM patients in terms of LVR after AMI. However, DM patients had a statistically significant increased risk of developing HF and valvular heart disease compared to non-DM patients after AMI.

## Introduction

Globally, diabetes mellitus (DM) is a serious health issue [1]. The prevalence of DM in the Kingdom of Saudi Arabia (KSA) in 2014 was estimated to be 13.4% [2]. By 2030, it is predicted that the KSA will be among the top five countries with the highest prevalence of type 2 DM, moving up from its current position as one of the top 10 countries with the highest incidence of DM [1]. The presence of DM has been found to be a strong indicator of poor cardiovascular outcomes, such as recurrent infarction and heart failure (HF), in individuals with acute myocardial infarction (AMI) [1-3]. According to multi-center registry research that included 50 Saudi hospitals, 52.7% of patients with AMI had DM [4].

Left ventricular remodeling (LVR) following AMI also plays a major role in the development of HF [5]. The progression to HF after AMI is affected by several factors, such as the location of the infarct and the size of the infarct [6]. However, the impact of DM on post-AMI LVR has shown mixed findings [7,8]. LVR is known as a compensatory mechanism that leads to structural and dysfunctional adaptations such as left ventricular dilation and reduced left ventricular ejection fraction (LVEF) following myocardial injury [8-10]. LVR, defined by an increase in the left ventricular end-systolic volume greater than 15%, has been observed in 30% of anterior myocardial infarction (MI) cases and nearly 17% of non-anterior MI cases given they underwent timely percutaneous coronary intervention (PCI) and were given cardioprotective medication such as angiotensin-converting enzyme inhibitors or angiotensin II receptor blockers, beta-blockers, mineralocorticoid receptor antagonists, and statins [11]. DM itself can cause LVR [12], and with regard to DM-related cardiomyopathy, LVR is only caused by DM [13]. The worse the LVR, the worse the long-term clinical outcomes and progression to HF. What remains unclear is whether ST-elevation myocardial infarction (STEMI) patients with concomitant DM suffer further increased LVR and subsequently worse clinical outcomes or not compared to non-DM patients. The primary objective of this study is to assess the association between LVR and DM in patients presenting with STEMI who had reperfusion therapy with optimal medical therapy after PCI.

## Materials and methods

Patients and study protocol

This is a retrospective cohort study that was conducted at King Faisal Cardiac Center in King Abdulaziz Medical City, National Guard Hospital, Jeddah, Saudi Arabia, during the period from January 2016 to December 2022. The patients' data were obtained from their medical records. The study included patients diagnosed with AMI who had undergone an echocardiogram at the time of admission (baseline) and at follow-up from six to 12 months after admission (mid-line). Patients who had other significant cardiac diseases or failed to complete the anticipated follow-up were excluded from the study. Patients who met the criteria were categorized into two groups: one comprising individuals with diabetes (DM) and the other consisting of individuals without diabetes (non-DM).

Data collection and variables

Patients were diagnosed with AMI based on the presence of symptoms indicative of AMI, elevated cardiac markers, and ST-segment elevation or depression on electrocardiography. Patients underwent echocardiography performed by a professional cardiac sonographer according to international guidelines. The collected data included demographics, clinical characteristics, echocardiographic parameters, and outcomes. Patient demographics and characteristics included age, gender, BMI, blood pressure, major cardiovascular events after AMI, and other comorbidities such as smoking, hypertension, dyslipidemia, and chronic kidney disease. Major cardiovascular events after MI were recorded as cardiopulmonary arrest, HF after STEMI, recurrent hospitalization, recurrence of MI, arrhythmia, valvular disease, stroke, or death. Other variables included medication at discharge, angiographic characteristics, and laboratory results. Variables used to assess the effect on cardiac remodeling included the difference in left ventricular ejection fraction (LVEF), left ventricular internal dimension in diastole (LVIDd), left ventricular internal dimension in systole (LVIDs), interventricular septum thickness (IVST), posterior left ventricular wall thickness (PWT), left ventricular mass index (LVMI), left atrial volume index (LAVI), relative wall thickness (RWT), and left ventricular end-diastolic volume index (LVEDVI) between the echocardiogram performed at admission and the follow-up visit.

Data analysis

The collected data were stored on a work computer accessed only by the authors. The data were analyzed using SPSS Statistics version 24 (IBM Corp. Released 2016. IBM SPSS Statistics for Windows, Version 24.0. Armonk, NY: IBM Corp.). Quantitative data was presented by mean and standard deviation, while qualitative data was presented by frequency and percentage. For comparing the quantitative data, the t-test was used. On the other hand, the Chi-squared test was used to compare qualitative data. A p-value of <0.05 was considered significant.

Ethical consideration

The study was approved by the ethical committee at King Abdullah International Medical Research Center with ethics approval number IRB/0281/23. Informed consent was not required due to the retrospective nature of this study.

## Results

Among the 171 individuals, 118 (69%) had DM. Table 1 compares the patient characteristics of the DM and non-DM groups. Similar initial characteristics were observed in both patients with and without DM. The mean age and BMI were comparable between the two groups. Male patients were more predominant than females in both groups. Additionally, DM patients were more likely to have a history of hypertension, dyslipidemia, smoking, and stress hyperglycemia. The DM group also had a higher hospitalization rate compared to the non-DM group. Moreover, laboratory results show that admission blood plasma glucose and hemoglobin A1c mean values were higher in the DM group. Treatment with insulin and/or oral hyperglycemic medications was used to treat 91 (53%) patients. About 27.97% of DM patients didn't use anti-diabetic medications. Most of the patients used dual antiplatelet therapy with a prevalence of 64%. Both DM and non-DM groups received antihypertensive medications such as angiotensin-converting enzyme inhibitors at discharge, with a prevalence of 84%.

**Table 1:** Patient’s characteristics, laboratory data, and medications between the DM and non-DM groups LDL: low-density lipoprotein, HDL: high-density lipoprotein, BNP: brain natriuretic peptide, BMI: body mass index, DM: diabetes mellitus

		All (N=171)	DM (N=118)	Non-DM (N=53)	p-value
Age, year (mean, SD)		63.5±12.9	65±11.2	60.2±15.8	0.026
Female sex, n (%)		27 (15.8)	23 (19.49)	4 (7.55)	0.048
Male sex, n (%)		144 (84.2)	95 (80.51)	49 (92.45)	
BMI, kg/m2 (mean, SD)		27.86±5.63	28.10±5.58	27.32±5.75	0.4
Hospitalizations (mean, SD)		2.70±2.23	3±2.48	1.98±1.22	0.012
Hypertension, n (%)		112 (65.5%)	93 (78.8%)	19 (35.8%)	<0.0001
Discharge heart rate (mean, SD)		80.85±12.14	79.38±11	81.54±14.36	0.29
Dyslipidemia, n (%)		46 (26.9)	38 (32.2)	8 (15.1)	0.02
DM, n		118	118	0	
Stress hyperglycemia, n (%)		119 (69.6)	103 (87.3)	16 (30.2)	<0.0001
Smoker		69 (40.4)	41 (34.7)	28 (52.8)	0.026
Systolic blood pressure on discharge, mmHg (mean, SD)		120.5±20.45	121.62±20.47	118±20.38	0.29
Laboratory data					
Hemoglobin A1C, % (mean, SD)		8.09±2.45	8.96±2.32	6.1±1.32	<0.0001
Admission blood plasma glucose, mmol/l (mean, SD)		12.35±6.96	14.54±7.09	7.5±3.3	<0.0001
Total cholesterol, mmol/l (mean, SD)		4.71±1.31	4.7±1.4	4.79±1.05	0.7
LDL cholesterol, mmol/l (mean, SD)		2.63±1.09	2.54±1.15	2.86±0.93	0.21
HDL cholesterol, mmol/l (mean, SD)		0.93±0.24	0.92±0.25	0.97±0.23	0.32
Triglyceride, mmol/l (mean, SD)		1.88±1.49	1.94±1.6	1.73±1.14	0.43
Creatinine, mmol/l (mean, SD)		115±108.2	130.2±127	81.28±17.5	<0.0001
BNP at admission, pg/ml (mean, SD)		308.6±605	361.9±697.8	188.1±275.4	0.006
Cardiac troponin at admission, ng/L (mean, SD)		14170.79±31409.168	10771.55±28684.73	21369.17±35760.6	0.015
Medication					
Antiplatelet	Single antiplatelet (n, %)	60 (35)	47 (39.83)	13 (24.53)	0.052
	Dual antiplatelet (n, %)	111 (64)	71 (60.17)	40 (75.47)	
Antihypertensive medications	Use of antihypertensive medication (n, %)	144 (84)	96 (81.36)	48 (90.57)	0.13
	No use of antihypertensive medication (n, %)	27 (15)	22(18.64)	5 (9.43)	
Antidiabetic medications	Use diabetes medication (n, %)	91(53)	85 (72.03)	6 (11.32)	<0.0001
	No use of diabetes medication or treatment (n, %)	80(47)	33 (27.97)	47 (88.68)	
Other cardiac medications	Use of other cardiac medications (n, %)	149 (87.13)	101 (85.59)	48 (90.57)	0.37
	No use of other cardiac medications (n, %)	22 (12.87)	17 (14.41)	5 (9.43)	

A comparison of the differences in angiographic features between the two groups is presented in Table 2. Although there was no statistically significant difference (p=0.253), both groups had a higher incidence of the left main trunk and/or left anterior descending artery affected. Moreover, both groups performed primary PCI in 78.4% of cases involving AMI. However, it was more frequently performed in the non-DM group than in the DM group (54.4% vs. 24.0%). In addition, DM patients had more diseased vessels compared to those without DM, although the difference was not statistically significant (p=0.063).

**Table 2:** Angiographic characteristics between the DM and non-DM groups PCI: percutaneous coronary intervention, CABG: coronary artery bypass grafting, DM: diabetes mellitus

Angiographic characteristics		All (N=171)	DM (N=118)	Non-DM (N=53)
Type of the first intervention	Primary PCI (n, %)	134/171 (78.4)	93/118 (54.4%)	41/53 (24)
	Thrombolytic therapy (n, %)	16/171 (9.4)	9/118 (5.3)	7/53 (4.1)
	CABG (n, %)	12/171 (7.0)	9/118 (5.3)	3/53 (1.8)
	Not determined (n, %)	9/171 (5.3)	7/118 (4.1%)	2/53 (1.2%)
Coronary artery affected	Left main trunk and/or left anterior descending artery (n, %)	102/171 (59.6)	67/118 (39.2)	35/53 (20.5)
	Left circumflex artery (n, %)	44/171 (25.7)	35/118 (20.5)	9/53 (5.3)
	Right coronary artery (n, %)	63/171 (36.8)	43/118 (25.1)	20/53 (11.7)
Number of diseased vessels	1 vessel (n, %)	63/171 (36.8)	37/118 (31.4)	26/53 (49.1)
	2 vessels (n, %)	29/171 (17)	21/118 (17.8)	8/53 (15.1)
	3 or more vessels (n, %)	50/171 (29.2)	35/118 (29.7)	15/53 (28.3)
	Not determined (n, %)	29/171 (17)	25/118 (21.2)	4/53 (7.5)

**Figure 1:**
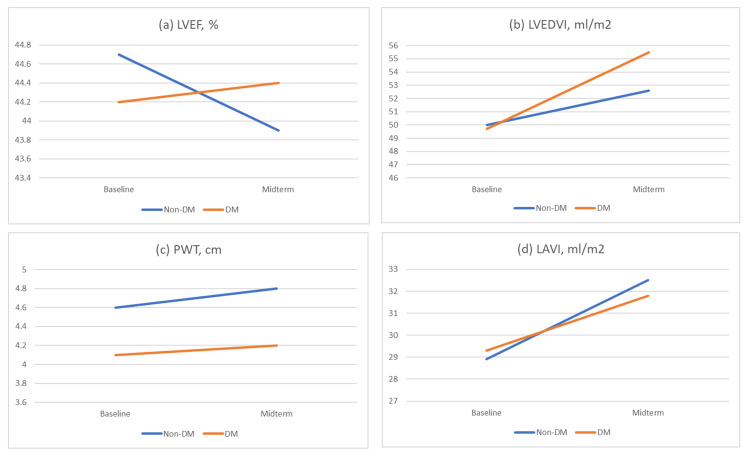
Comparison of temporal changes in LVEF (a), LVEDVI (b), PWT (c), and LAVI (d) LVEF: left ventricular ejection fraction, LVEDVI: left ventricular end-diastolic volume index, PWT: posterior left ventricular wall thickness, LAVI: left atrial volume index, DM: diabetes mellitus

Table 3 summarizes the echocardiographic findings of patients with and without DM at baseline and mid-term follow-up, along with the p-values for comparison. The results in Table 3 compare the echocardiographic findings between the two groups, demonstrating that there were no significant differences in the changes of LVEF from baseline to midterm between the DM group (0.2%) and non-DM group (-0.3%; p=0.884). The changes in LVEDVI from baseline to midterm were not significant between the DM (5.8%) and non-DM groups (2.6%; p=0.913). Figure 1 illustrates the temporal change in LVEF, LVEDVI, PWT, and LAVI, comparing the baseline and mid-term follow-up. The DM group exhibited greater changes in LVEDVI compared to the non-DM group at both time points. Additionally, the LVEF was initially lower in the DM group than in the non-DM group. While the PWT was higher in the non-DM group, the temporal changes in PWT and LAVI between the two groups were similar from baseline to mid-term follow-up. The p-values for these parameters were greater than 0.05, suggesting that there is no strong evidence to support a significant difference between the two groups.

**Table 3:** Echocardiographic features at baseline and mid-term in the DM and non-DM groups LVEF: left ventricular ejection fraction, LVDd: left ventricular internal dimension in diastole, LVIDs: left ventricular internal dimension in systole, IVST: interventricular septum thickness, PWT: posterior left ventricular wall thickness, LVMI: left ventricular mass index, LAVI: left atrial volume index, RWT: relative wall thickness, LVEDVI: left ventricular end-diastolic volume index, DM: diabetes mellitus

Echocardiographic features	DM (N=118)		Non-DM (N=53)		p-value
	Baseline	Midterm	Baseline	Midterm	
LVEF, % (mean, SD)	44.2±8.6	44.4±8.5	44.7±8.7	43.9±9.1	0.884
LVDd, cm (mean, SD)	16.7±18.4	17.7±19.2	19.2±9.6	20.7±9.2	0.903
LVIDs, cm (mean, SD)	12.6±14.0	13.6±15.2	14.1±15.4	15.3±15.1	0.430
IVST, cm (mean, SD)	4.3±4.5	4.1±4.4	4.6±4.8	4.9±4.8	0.698
PWT, cm (mean, SD)	4.1±4.3	4.2±4.4	4.6±4.7	4.8±4.7	0.996
LVMI, g/m2 (mean, SD)	82.9±17.4	85.7±24.3	83.6±20.2	87.0±31.7	0.993
LAVI, ml/m2 (mean, SD)	29.3±6.9	31.8±8.5	28.9±8.9	32.5±10.0	0.659
RWT (mean, SD)	0.43±0.08	0.42±0.11	0.46±0.11	0.43±0.09	0.099
LVEDVI, ml/m2 (mean, SD)	49.7±11.3	55.5±16.4	50.0±17.4	52.6±14.0	0.913

## Discussion

DM has been a serious global and local health issue, affecting 13.4% of the population in Saudi Arabia [2]. DM has generally been a strong poor prognostic factor for cardiovascular diseases, increasing the risk of HF and recurrent infarctions in patients with AMI [1-3]. One of the possible mechanisms by which DM has been a poor prognostic factor is through the induction of LVR, in which the left ventricle (LV) undergoes structural and dysfunctional adaptations as a response to AMI [11]. As the LVR becomes worse, there is an increased risk of long-term clinical outcomes and progression to HF. What remains uncertain is whether patients with AMI and DM suffer increased LVR and subsequently worse clinical outcomes or not compared to non-DM patients. The primary aim of this study is to compare the effect of cardiac remodeling on patients with DM and those without DM who were diagnosed with AMI.

Among the 171 patients included in the study, 118 (69%) had DM. The two groups of the study had similar baseline characteristics in terms of mean age and BMI; however, there were baseline differences in terms of gender, comorbidities such as hypertension, dyslipidemia, and smoking, and laboratory values upon admission such as hemoglobin A1C and blood plasma glucose. In regard to angiographic characteristics, PCI was the predominant intervention in both groups, with 78% (n=93) in the DM group and 77% (n=41) in the non-DM group. In terms of the number of diseased vessels, no difference was found between the two groups, and half of the participants had two or three vessels that affected 46.1% (n=79). As for the primary aim of the current study, which was to investigate LVR between DM and non-DM patients, the study results showed no significant difference between the two groups in terms of echocardiographic features at baseline and mid-term after 6-12 months.

LVEF in the DM group changed by +2.2% from baseline, while in the non-DM group, the change was -0.8% from baseline without a statistically significant difference. This finding supports the conclusion of Solomon et al. that DM patients had a milder decline in ejection fraction following MI when compared to non-DM patients [14]. In addition, the increase in LVEDVI was found to be +5.8 ml and +2.6 ml in DM and non-DM, respectively, though this change was not statistically significant. This contradicts Yap et al.'s conclusion that DM influences cardiac remodeling leading to left ventricular stiffness and concentric hypertrophy with HF with preserved ejection fraction (HFpEF) and eccentric with HF with reduced ejection fraction (HFrEF). This difference in results might be secondary to a relatively inadequate sample size [15]. Other echocardiographic features and BNP levels at baseline and mid-term were comparable and similar in the two groups without a statistically significant difference, which is different from what Ephysten et al. concluded regarding BNP, finding that DM is associated with a further increase in BNP possibly secondary to LV strain [16].

Moreover, with respect to cardiovascular events and clinical outcomes, both DM and non-DM patients had similar outcomes in terms of mortality, recurrent hospitalization, recurrent AMI, and risk of arrhythmia. However, DM patients had a statistically significant increased risk of developing HF and valvular heart disease compared to non-DM patients after AMI. The increased risk of developing HF concurs with the findings of Mao et al., suggesting that patients with DM have a greater risk of developing HF [17]. Furthermore, an increased incidence of valvular heart disease has been reported in diabetics. A study by Freeman et al. demonstrated that DM accelerates the progression of aortic stenosis through increased inflammation, lipid accumulation, and subsequent dystrophic calcification [18]. Moreover, diabetics are prone to both accelerated functional mitral regurgitation and mitral stenosis [19,20].

Study limitations

While this study provides valuable insights into the relationship between DM and LVR in AMI patients, it is important to acknowledge its limitations. One of the limitations is the relatively small sample size, which may have affected the statistical power and generalizability of the findings. However, despite this limitation, the study was still able to identify trends and patterns that contribute to the existing body of knowledge in this field. Another limitation is the presence of baseline differences between the two groups, such as gender, comorbidities, and laboratory values. Nevertheless, these differences were acknowledged in the analysis, and appropriate adjustments were made to account for their potential influence on the results. Additionally, the study relied on echocardiographic assessments, which were obtained from old records spanning over a period of six years and collected by different operators using potentially different machines, which may introduce limitations in accurately capturing the complete extent of LVR. However, it is worth acknowledging that despite these limitations, echocardiography remains a widely utilized and reliable method for evaluating cardiac structure and function. However, echocardiography remains a widely used and reliable method for evaluating cardiac structure and function. Lastly, the follow-up duration was limited to mid-term, which may not have captured long-term clinical outcomes and the progression of HF. Despite this limitation, the study provides important insights into the intermediate-term effects of DM on LVR. Overall, while this study has its limitations, the findings contribute to the existing literature and warrant further investigation with larger sample sizes, longer follow-up periods, and a multimodal approach to cardiac assessment.

## Conclusions

This retrospective study aimed to investigate the effect of DM on cardiac remodeling and clinical outcomes in patients with AMI. The study included 171 individuals, of whom 69% had DM. The findings revealed that patients with DM had similar baseline characteristics compared to non-DM patients. The echocardiographic parameters, including LVEF and LVEDVI, did not show significant differences between the two groups at baseline and mid-term follow-up. It is worth noting that the presence of DM was associated with a higher incidence of comorbidities, hospitalization, and stress hyperglycemia. However, the study did not find strong evidence to support a significant association between DM and cardiac remodeling or clinical outcomes in patients with AMI. These findings emphasize the need for comprehensive management strategies that address both DM and AMI to optimize long-term clinical outcomes. Further research is warranted to explore the complex relationship between DM and cardiac remodeling in larger prospective studies.
